# Targeted delivery of quercetin by biotinylated mixed micelles for non-small cell lung cancer treatment

**DOI:** 10.1080/10717544.2022.2055225

**Published:** 2022-03-28

**Authors:** Kangkang Li, Xinlong Zang, Xiangjun Meng, Yanfeng Li, Yi Xie, Xuehong Chen

**Affiliations:** a School of Basic Medicine, Qingdao University,Qingdao, China; b Qingdao Mental Health Center,Qingdao, China

**Keywords:** Mixed micelles, biotin, NSCLC, targeted delivery, quercetin

## Abstract

Lung cancer is the leading cause of cancer death world-wide and its treatment remains a challenge in clinic, especially for non-small cell lung cancer (NSCLC). Thus, more effective therapeutic strategies are required for NSCLC treatment. Quercetin (Que) as a natural flavonoid compound has gained increasing interests due to its anticancer activity. However, poor water solubility, low bioavailability, short half-life, and weak tumor accumulation hinder *in vivo* applications and antitumor effects of Que. In this study, we developed Que-loaded mixed micelles (Que-MMICs) assembled from 1,2-distearoyl-sn-glycero-3-phosphoethanolamine–poly(ethylene glycol)–biotin (DSPE–PEG–biotin) and poly(ethylene glycol) methyl ether methacrylate–poly[2-(dimethylamino) ethyl acrylate]–polycaprolactone (PEGMA–PDMAEA–PCL) for NSCLC treatment. The results showed that Que was efficiently encapsulated into the mixed micelles and the encapsulation efficiency (EE) was up to 85.7%. Cellular uptake results showed that biotin conjugation significantly improved 1.2-fold internalization of the carrier compared to that of non-targeted mixed micelles. *In vitro* results demonstrated that Que-MMICs could improve cytotoxicity (IC_50_ = 7.83 μg/mL) than Que-MICs (16.15 μg/mL) and free Que (44.22 μg/mL) to A549 cells, which efficiently induced apoptosis and arrested cell cycle. Furthermore, Que-MMICs showed satisfactory tumor targeting capability and antitumor efficacy possibly due to the combination of enhanced permeability and retention (EPR) and active targeting effect. Collectively, Que-MMICs demonstrated high accumulation at tumor site and exhibited superior anticancer activity in NSCLC bearing mice model.

## 1. Introduction

Lung cancer is the leading cause of cancer death world-wide, with approximately 2.1 million new cases and 1.8 million deaths every year (Bray et al., 2018). Non-small cell lung cancers (NSCLC) are predominant and account for 80–85% in all lung cancer. The prognosis of NSCLC is very poor and 5-year survival rate is 15% for NSCLC patients (Kris et al., 2014). To this study, chemotherapy remains a main treatment option for NSCLC (Jalal et al., 2009; Yu et al., 2015). Although chemotherapeutics clinically available improve the outcomes in patients with NSCLC, the inevitable prevalence of drug resistance and unbearable side effects generally lead to incomplete and temporary antitumor effects of these agents, which call for new and safe drugs (Chen et al., 2018).

Numerous nature compounds have shown high efficacy and less toxicity, and attracted increasing interests in chemoprevention and chemotherapy (Senft et al., 2010). Quercetin (3′,3′4′,5,7-pentahydroxyflavone) (Que) is widely present in human diets, such as pear, onion, apple, and crataegus, etc. Que is considered as a promising therapeutic and preventive agent due to its good anti-inflammatory, antioxidant, antimicrobial, anticancer, and free-radical scavenging properties without significant cytotoxicity (Liu et al., 2006; Cai et al., 2015; Mukherjee & Khuda-Bukhsh, 2015; Jiang et al., 2016). Therefore, Que has been extensively explored in cancer, cardiovascular and metabolic diseases. In antitumor treatment, Que has been shown satisfactory antitumor efficiency through inducing apoptosis, arresting cell cycle, reducing angiogenesis and metastasis, reversing drug/radiotherapy resistance, and modulation tumor immunity (Hirvonen et al., 2001; Nair et al., 2015; Zang et al., 2021). For NSCLC, Que can directly suppress the growth of tumor cells as an aurora B inhibitor (Xingyu et al., 2016). Que significantly suppressed lung cancer cells invasion and migration through reduction in metalloproteinases (MMPs) expression and activity in a dose-dependent manner (Chuang et al., 2016). Furthermore, Que has shown the potential as a chemosensitizer and immune modulator to improve antitumor efficacy in lung cancers in combination with other therapies (Cincin et al., 2014; Lee et al., 2015).

In spite of the excellent antitumor effects, low water solubility, poor metabolic stability and bioavailability, short half-life and poor tumor targeting capability have been proven almost to be an insurmountable obstacle for clinical translations of Que (Dabeek & Marra, 2019). Recent progresses in nanotechnology offer more possibilities to overcome these drawbacks of drugs themselves, which can efficiently encapsulate them and ameliorate their pharmacokinetics and pharmacodynamic profiles (Zhou et al., 2020; Li et al., 2021a,b; Zhang et al., 2022). Interestingly, nanoparticles are capable of passively accumulating into tumor sites as a result of incomplete vessels and lymphatic blockage termed as enhanced permeability and retention (EPR) effect (Fang et al., 2011). Moreover, ligands, peptides, and proteins modification enable active targeting to tumor cells, stromal and even subcellular organelle (Zheng et al., 2019; Zhang et al., 2020). The mentioned properties confer nanomedicines the capacity to improve therapeutic efficiency and minimize off-targets effects. To enable more distribution of Que into tumor, versatile nanoplatforms have been developed to improve its solubility in water, bioavailability, and tumor-targeting ability (Liao et al., 2021). For example, Que-loaded nanomicelles assembled from DSPE-PEG showed superior antitumor efficacy with decreased tumor proliferation rate compared to free Que in the PC-3 xenograft mouse model (Zhao et al., 2016). Furthermore, mixed micelles with two or more copolymers have demonstrated a higher drug loading capacity, stronger stability, prolonged release, and better bioavailability (Xu et al., 2016; Cagel et al., 2017; Jin et al., 2018). For instance, the mixed micelles composed of DSPE-PEG and d-α-tocopherol polyethylene glycol succinate (TPGS) indicated better stability than that of either DSPE-PEG or TPGS alone (Zhang et al., 2019).

**Figure 1.**
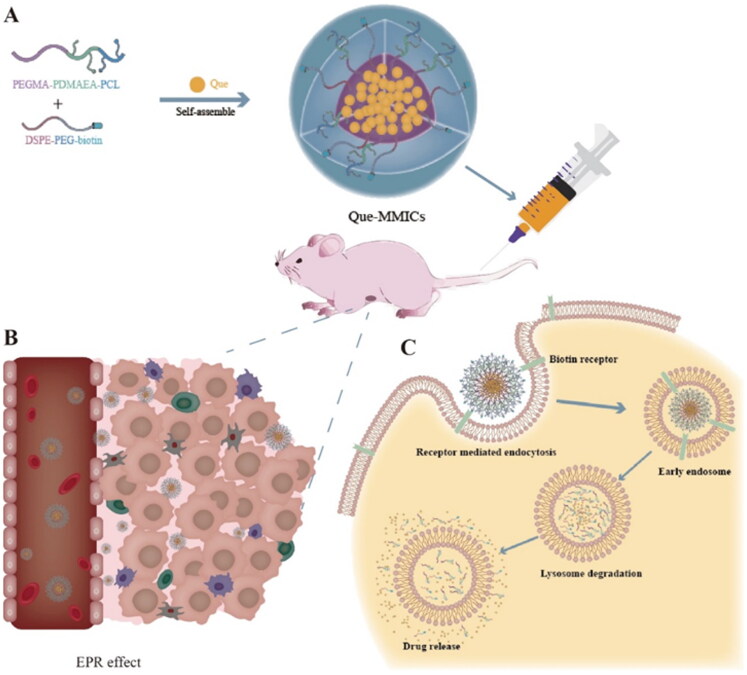
(A) The micelles were self-assembled driven by the hydrophobic interactions between hydrophobic segments of polymer and Que. (B) Higher accumulation in tumor region after intravenous administration through EPR effect and biotin mediated active targeting. (C) Schematic illustration of the intracellular trafficking of micelles.

In this work, we developed a favorable delivery system based on mixed micelles to carry Que for NSCLC treatment and amplify its antitumor efficacy. The Que-loaded mixed micelles (Que-MMICs) were composed of the amphiphilic triblock copolymer of poly(ethylene glycol) methyl ether methacrylate–poly[2-(dimethylamino) ethyl acrylate]–polycaprolactone (PEGMA–PDMAEA–PCL) and 1,2-distearoyl-sn-glycero-3-phosphoethanolamine–poly(ethylene glycol)–biotin (DSPE–PEG–biotin). The hydrophobic inner core of Que-MMICs was entrapped with water-insoluble Que meanwhile the hydrophilic outer shell was to hide the inner core, which improved stability and prolong circulation time. Biotin receptors are upregulated in lung cancer cells, which could serve as a useful biomarker for potential tumor-targeting therapy (Maiti et al., 2013; Yan et al., 2019). Thus, biotin was introduced into the micelles to facilitate their accumulation in tumor via the receptor-mediated endocytosis. Herein, the synthesis, preparation, and characterization of the resultant micelles were described and the targeting and antitumor efficacy was thoroughly investigated *in vitro* and *in vivo*. The results implied that Que-MMICs could effectively inhibit tumor cells, promote their apoptosis and cycle arrest *in vitro* meanwhile suppress tumor growth and reduce angiogenesis in A549 tumor bearing nude mice, which was a promising strategy for lung cancer treatment (Figure 1).

## 2. Materials and methods

### 2.1. Materials

All reagents were obtained from commercial sources: dodecanethiol, potassium phosphate, carbon disulfide, 4-(chloromethyl)benzyl alcohol, 6-hexanolactone, Tin(II) 2-ethylhexanoate, 2-(dimethylamino)ethyl acrylate (DMAEA), poly(ethylene glycol)methyl ether methacrylate (PEGMA, 500 Da), 2,2′-azobis(2-methylpropionitrile) (AIBN, 99%) and Nile red (NLR) were purchased from Energy Chemical (Shanghai, China). 1,2-Distearoyl-sn-glycero-3-phosphoethanolamine–poly(ethylene glycol)–biotin was purchased from Guangzhou Tansh (Guangzhou, China). Immunohistochemistry (IHC) detection system kit and antibodies were purchased from Bioss (Beijing, China). DiR was purchased from MeilunBio (Suzhou, China). MTT solution, 2% phosphotungstic acid and 4′,6-diamidino-2-phenylindole (DAPI) were obtained from Solaibio (Shanghai, China). One Step TUNEL Apoptosis Assay Kit and Hematoxylin and Eosin Staining Kit were purchased from Beyotime (Shanghai, China). All other chemical regents were obtained from Sinopharm Chemical Reagent Co., Ltd. (Shanghai, China).

### 2.2. Mice and cell line

Male nude Balb/c mice with body weight of 18–22 g were obtained from Sibeifu (Beijing, China) and kept under specific pathogen-free conditions at 23 ± 2 °C with water and food given ad libitum. Experiments and care/welfare were in agreement with a protocol approved by the Qingdao University Animal Studies Committee that approved with all our experiments.

A549 cells were purchased from ATCC (Manassas, VA) and cultured in RPM1640 (Cienry, Zhejiang, China) supplemented with 10% FBS (Omni, Houston, TX) and 1% penicillin/streptomycin (Cienry, Zhejiang, China) at 37 °C in a humidified atmosphere with 5% CO_2_.

### 2.3. Synthetic protocols

#### 2.3.1. Synthesis of RAFT/ROP initiator

The dual ring-opening polymerization (ROP)/reversible addition fragmentation chain transfer (RAFT) initiator CTA was prepared as previously reported (Petzetakis et al., 2013). Briefly, dodecanethiol (6.46 g, 31.9 mmol), potassium phosphate (7.45 g, 35.1 mmol), and carbon disulfide (5.95 mL, 99.0 mmol) were dissolved into acetone (600 mL) and the reaction was performed at ambient temperature for 2 h. Subsequently, 4-(chloromethyl)benzyl alcohol (5 g, 31.9 mmol) was added and the mixture was further stirred for another 23 h. After removing the solvent under vacuo, the resultant solid was re-dissolved in dichloromethane (CH_2_Cl_2_) (200 mL) that was sequentially washed with HCl (1 M, 150 mL × 2), water (150 mL × 3), brine (150 mL), and then dried using anhydrous MgSO_4_. The solvent was removed in vacuo to give the crude product as a yellow solid. Washing with hexanes gave the product as a yellow solid (11.45 g, 90%). This product was dried under a P_2_O_5_ atmosphere in a desiccator for two days and stored under N_2_ atmosphere. ^1^H NMR spectra of CTA were recorded on AVANCE III HD 400 MHz (Bruker, Fällanden, Switzerland).

#### 2.3.2. Synthesis and characterization of CTA-PCL

CTA-PCL was synthesized via ROP reactions (de Freitas et al., 2013). First, Sn(Oct)_2_ (0.05 g, 0.12 mmol) was dissolved in 1 mL CH_2_Cl_2_. Mixture of dried 6-hexanolactone (5.0 g, 21.9 mmol), Sn(Oct)_2_, and CTA (0.1 g, 0.25 mmol) were added into a clean and dry polymerization flask, and then vacuum-pumping and argon-filling were alternately performed three times to remove oxygen and CH_2_Cl_2_ in the flask. The solution was stirred under the protection of nitrogen atmosphere at 120 °C for 24 hours. After cooling to room temperature, the crude product was dissolved in CH_2_Cl_2_ and purified with precipitation in cooled diethyl ether for three times. Finally, CTA-PCL was dried at room temperature in vacuum until constant weight (4.03 g, 79%). ^1^H NMR spectra of CTA-PCL were recorded on AVANCE III HD 400 MHz (Bruker, Fällanden, Switzerland). ^1^H NMR spectra of CTA-PCL were recorded on AVANCE III HD 400 MHz (Bruker, Fällanden, Switzerland).

#### 2.3.3. Synthesis and characterization of PEGMA–PDMAEA–PCL

The block copolymers were synthesized by RAFT copolymerization of EGMA, DMAEA, and CTA-PCL served as macroinitiator. CTA-PCL (0.3 g, 0.03 mmol), PEGMA (0.2 g, 0.4 mmol), DMAEA (0.05 g, 0.7 mmol), and AIBN (0.005 g, 0.03 mmol) were dissolved in 2 mL anhydrous 1,4-dioxane and sealed in a flask under the protection of nitrogen. The reaction was proceeded under 70 °C for 24 h. The solution was then cooled down to room temperature and purified with precipitation in cooled diethyl ether for three times. Finally, PEGMA–PDMAEA–PCL was dried at room temperature in vacuum until constant weight (0.46 g, 83.6%). ^1^H NMR spectra of PEGMA–PDMAEA–PCL were recorded on AVANCE III HD 400 MHz (Bruker, Fällanden, Switzerland).

### 2.4. Hemolysis test

To assess the potential of PEGMA–PDMAEA–PCL for application *in vivo* (e.g. intravenous injection), the hemocompatibility was evaluated through a hemolysis approach. Briefly, 2 mL of fresh rabbit blood was added in a heparin-containing anticoagulant tube, which was diluted by PBS at a final concentration of 10% red blood cells (RBC) before use. The micelles of PEGMA–PDMAEA–PCL with different concentrations were incubated with equivalent RBCs for 4 h in a 37 °C water bath followed by centrifugation (2000 rpm, 5 min). The absorbance of the supernatant containing the released hemoglobin (Hb) was then measured at 540 nm using the spectrophotometric plate reader (SynergyMx, BioTek, Winooski, VT). The hemolysis percentage was then calculated used the following equation:

$$\text{Hemolysis percentage }(\%)\;=\frac{A_{s}-A_{n}}{A_{p}-A_{n}}\times 100\%$$

where *A_s_*, *A_p_*, and *A_n_* represent the absorbance of RBCs solutions incubated with micelles samples, distilled water and PBS, respectively.

### 2.5. Preparation and characterization of Que-MMICs nanoparticles

Thin-film hydration method was applied to prepare Que-loaded PEGMA–PDMAEA–PCL/DSPE–PEG–biotin mixed micelles (Que-MMICs). In brief, 8 mg PEGMA–PDMAEA–PCL and 2 mg DSPE–PEG–biotin were dissolved in 6 mL acetonitrile which was mixed with 0.5 mL methanol solution containing 2 mg/mL Que. The solvent was removed using a rotary evaporator at 40 °C. The resultant thin film was hydrated with 2 mL of PBS solution for 30 min at 60 °C to obtain a micellar solution. Unentrapped Que was removed by filtration through a 0.22 μm filter. The nontargeted micelles (Que-MICs) were prepared using the same method in the absence of DSPE–PEG–biotin. The preparation of DiR or NLR labeled micelles (DiR-MMICs, NLR-MMICs) was also prepared using the same protocol mentioned above except that DiR or NLR was used to replace Que in the mixtures.

The particle diameter of different formulations was measured via dynamic light scattering (DLS) technique using a Zetasizer Nano-ZS (Malvern Instruments, Worcestershire, UK). All results were the mean of three test runs. The morphology was observed using JEM-2100 transmission electron microscopy (TEM, JEOL, Tokyo, Japan). Briefly, the sample was added onto a copper mesh and stained with 2% phosphotungstic acid, followed by observation under JEM-2100 TEM (JEOL, Tokyo, Japan). The *in vitro* stability profile of micelles was evaluated by particle diameter. The micelles were stored at room temperature (25 °C) for 72 h and the particle diameter was measured at different intervals.

The encapsulation efficiency (EE) and drug loading efficiency (DL) of the mixed micelles were measured using UV absorbance method (Kumar et al., 2009). In brief, the micelles were dissolved or swelled in methanol, and then the loaded drug Que was measured at 372 nm with a NanoPhotometer UV-vis spectrophotometer (IMPLEN, München, Germany). The EE and DL in the micelles were calculated using the following formulas:

$$\mathrm{EE}\;(\%)=\;\frac{W_{\mathrm{Que}}}{W_{\mathrm{Total}\;\mathrm{Que}}}\;\times \;100\;\%$$

$$\mathrm{DL}\;(\%)=\;\frac{W_{\mathrm{Que}}}{W_{\mathrm{Micelles}}}\;\times \;100\;\%$$

where *W_Que_*, *W_total Que_*, and *W_Micelles_* are the weight of loaded Que, total Que, and the micelles, respectively.

The *in vitro* release profile of mixed micelles was evaluated using a dialysis bag (molecular weight cutoff, 3500 Da) against PBS in a shaking incubator (37 °C, 100 rpm). In brief, 1 mL of Que-MMICs was introduced into a dialysis bag and further immersed in 50 mL fresh PBS containing 0.2% Tween 80 to mimic ‘sink conditions.’ At different intervals, 1 mL aliquot was taken out and replenished with an equal volume of fresh medium. The concentration of Que in the release medium was determined using a UV–vis spectrophotometer at 372 nm, as described above.

### 2.6. Cellular uptake assay

Cellular uptake of NLR-MMICs in A549 cells was measured using confocal laser scanning microscopy (CLSM) imaging and flow cytometry. A549 cells were seeded in six-well plate containing glass coverslips at a density of 2 × 10^5^ cells per well and further cultured in RPM1640 containing 10% FBS and 1% penicillin–streptomycin for 24 h. NLR-MMICs were then added and incubated for 6 h. After washing with PBS (pH 7.4), tumor cells were fixed with 4% formaldehyde for 0.5 h and the nucleus was stained by DAPI. CLSM images were captured using a TCS SP8 confocal microscope (Leica, Wetzlar, Germany).

For flow cytometry analysis, 5 × 10^5^ A549 cells were seeded into a six-well plate per well and cultured within complete medium for 24 h. NLR-MMICs were added and incubated with tumor cells for 1, 2, 4, and 6 h. These cells were washed with cold PBS (pH 7.4), trypsined and harvested following analysis by CytoFlex S flow cytometry (Beckman, Villepinte, France).

### 2.7. *In vitro* cell cytotoxicity

*In vitro* cytotoxicity of free Que and nanoformulations was investigated using the standard MTT assay. Briefly, 5 × 10^3^ A549 cells were seeded in 96-well plates in with complete RPM1640 medium (10% FBS) at 37 °C in a humidified 5% CO_2_ incubator for 24 h. After medium removal, free Que and nanoformulations were added and further incubated at 37 °C for 48 h. MTT solution (10 μg/mL) was added and incubated for 4 h followed by the addition of DMSO to dissolve the formazan crystals. The absorbance at 490 nm was recorded with a spectrophotometric plate reader (SynergyMx, BioTek, Winooski, VT). The cell viability was calculated using the following formula and IC_50_ values were calculated through GraphPrism software (San Diego, CA).

$$\text{Cell viability }(\%)\;=\frac{A_{\mathrm{drug}}-A_{\mathrm{blank}}}{A_{\mathrm{control}}-A_{\mathrm{blank}}}\times 100\%$$

where *A_Drug_* and *A_Control_* denote the absorbance in the presence and absence of different micelles formulations, respectively; meanwhile, *A_Blank_* denotes the absorbance of the blank culture medium.

### 2.8. Apoptosis and cell cycle analysis

Apoptosis and cell cycle were investigated using Annexin V-FITC-PI and PI staining followed by flow cytometry, respectively. Briefly, 3 × 10^5^ A549 cells were seeded in six-well plates 24 h prior to treatment with micelles containing 5 µg/mL and 10 µg/mL Que or with relevant controls in serum free culture medium. After treatment for 24 h as described above, both adherent and suspension cells were harvested, stained with PI and Annexin V-FITC Apoptosis Detection Kit (BD, Franklin Lakes, NJ) according to the manufacturer’s instructions, and analyzed by CytoFlex S flow cytometer (Beckman, Villepinte, France).

As for cell cycle assay, tumor cells treated with free Que and formulations for 24 h were trypsined, harvested and fixed in 70% ice cold ethanol at 4 °C for 12 h. Next, these cells were washed with PBS and stained with PI at room temperature for 0.5 h. The cells cycle distribution was determined and analyzed by flow cytometry as described above.

### 2.9. Wound-healing assay

A549 cells were seeded in 12-well plates, and grown overnight to reach a monolayer of confluence. The cells were wounded by scratching with pipet tips and washed twice by PBS to remove the non-adherent cells. Fresh RPM1640 medium containing different concentrations of drugs was added, and the cells were incubated for 48 hours. Images were taken with an inverted microscope Eclipse Ts2 (Nikon, Tokyo, Japan).

### 2.10. *In vivo* biodistribution in A549 tumor xenograft nude mice

In order to establish the model of A549 tumor xenograft nude mice, the mice were injected subcutaneously into right oxter with 0.1 mL PBS containing 1 × 10^6^ A549 cells. Subsequently, DiR-labeled micelles were administrated through the tail vein when the tumor volume reached to 100 mm^3^. At designed intervals, the mice were observed and imaged with IVIS Lumina XRMS III Image System (PerkinElmer, Waltham, MA). Main organs and tumor tissues were harvested and the images were captured after mice sacrifice at post 48 h injection.

### 2.11. *In vivo* antitumor efficacy in A549 tumor bearing mice

The mice model bearing A549 tumor was established as mentioned above. Once tumor volumes were above 50 mm^3^, these mice were randomly divided into four groups: (1) 8 mg/kg Que-MMICs, (2) 8 mg/kg Que-MICs, (3) 8 mg/kg Que, and (4) saline (5 mice per group). Que and reference formulations were then administrated every two days via tail intravenous injection. The body weight, tumor diameters, and appearance were monitored every two days. Tumor size was calculated by the formula: tumor volume=(*L*×*W*^2^)/2, where *L* is the longest diameter meanwhile *W* is the shortest. At post 11 days after the administration, mice were sacrificed after anesthesia with chloral hydrate meanwhile tumor tissues were dissected, harvested, and weighted followed by immersion into 4% paraformaldehyde for further histopathological investigation. The tumor growth inhibition (TGI) was calculated by the following equation:

$$\mathrm{TGI}\;(\%)=\frac{{{(V}_{t}/V_{0})}_{\mathrm{tested}\;\mathrm{group}}}{{{(V}_{t}/V_{0})}_{\mathrm{saline}\;\mathrm{group}}}$$

where *V_t_* and *V*_0_ denote the tumor volume at the beginning and ending, respectively.

### 2.12. Immunohistochemistry and TUNEL assay

All tumor tissues obtained above were embedded in paraffin and cut into 4 µm sections for IHC and HE staining. Continuous sections were deparaffinized in xylene, rehydrated in gradient ethanol, and immersed in deionized water. Antigen retrieval was performed in 0.01 M sodium citrate antigen-repair buffer and heated in microwave oven for 15 minutes. Tumor sections were subsequently quenched with 3% hydrogen peroxide in methanol (endogenous peroxidase blocker) for 30 minutes and blocked with 5% bovine serum albumin (BSA) for 15 minutes. These samples were incubated with primary antibodies against VEGF, MMP9, and CD31 overnight, followed by incubation with secondary antibodies. Subsequently, 3,3′-diaminobenzidine tetrachloride (DAB) was used to visualize the samples. For the detection of apoptotic cell death, tumor sections were stained with a One Step TUNEL Apoptosis Assay Kit (Beyotime, Shanghai, China) according to the manufacturer's instructions. Tumor and main organ sections were also counterstained with hematoxylin and eosin (H&E). These sections were observed with Eclipse Ts2 microscope (Nikon, Tokyo, Japan).

### 2.13. Statistical analysis

All data were expressed as the means ± SD from three independent experiments. Statistics was performed with Prism 5.0 statistical analysis software (La Jolla, CA). After normality tests, the mean differences of groups were assessed with one-way analysis of variance (one-way ANOVA), followed by post hoc Student–Newman–Keuls test. All statistical tests were two-sided, and *p*<.05 was considered to have significance. Calculated *p* values of *p*<.05, *p*<.01, and *p*<.001 were as indicated.

## 3. Results and discussion

### 3.1. Synthesis of the PEGMA–PDMAEA–PCL

**Figure 2.**
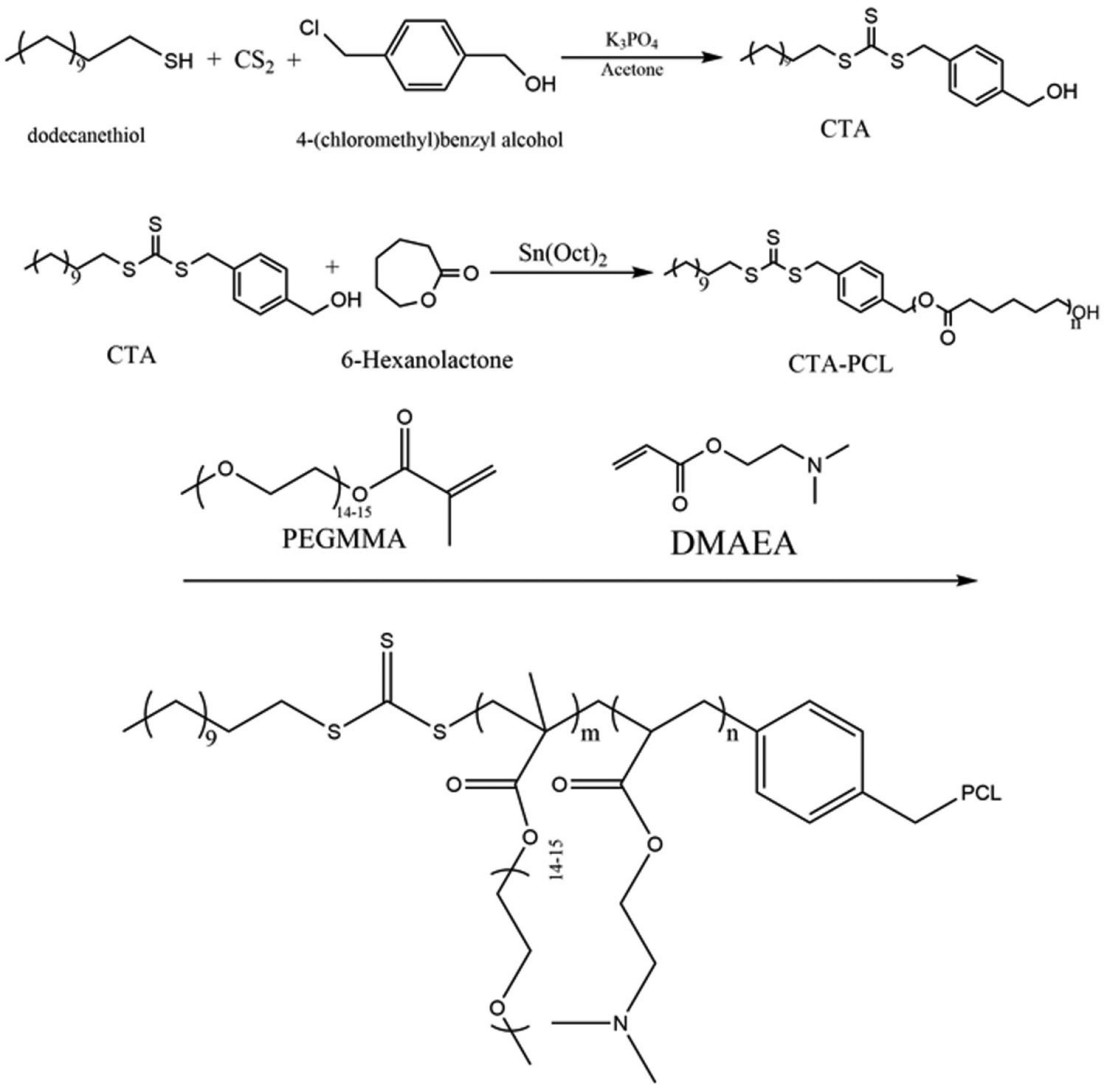
Synthesis route of PEGMA–PDMAEA–PCL.

**Figure 3.**
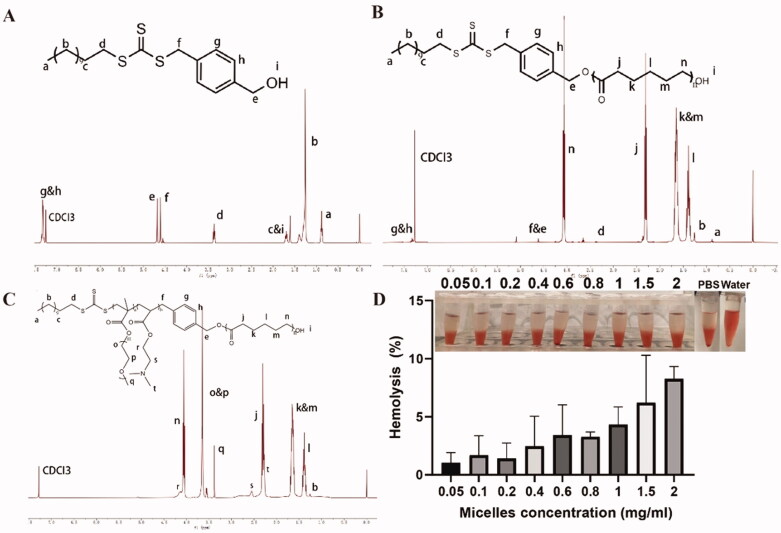
^1^H NMR spectra of (A) CTA, (B) CTA-PCL, and (C) PEGMA–PDMAEA–PCL. (D) Hemolysis behaviors of red blood cells treated with water (positive control), PBS (negative control), and PEGMA–PDMAEA–PCL solution at concentration varying from 0.05 to 2.00 mg/mL (mean ± SD, *n* = 3). The inset is a photograph of the corresponding samples.

We used a combination of ROP and RAFT polymerization to synthesize PEGMA–PDMAEA–PCL amphiphile copolymer. The synthesis route and typical ^1^H NMR spectra of the PEGMA–PDMAEA–PCL block copolymers are illustrated in Figures 2 and 3. First of all, ROP/RAFT initiator CTA was synthesized as previously reported (Petzetakis et al., 2013). As shown in Figure 3(A), peak at 7.30–7.20 ppm and 1.46–1.20 ppm belonged to benzene ring and –CH_2_– of b, which confirmed its successful synthesis. Subsequently, the CTA-PCL initiator was synthesized via ROP of 6-hexanolactone. Triple peaks at 4.1 and 2.3 ppm and multiple peaks at 1.7 and 1.4 ppm belonged to PCL. Finally, PEGMA–PDMAEA–PCL copolymer was synthesized through RAFT using trithiocarbonate as an initiator and its chemical structures was confirmed by ^1^H NMR. As shown in Figure 3(C), peak at 3.7 ppm belonged to –CH_2_CH_2_O– of PEGMA, and typical peaks of PDMAEA were shown at 2.6 ppm, which confirmed its successful synthesis. In addition, the number average molecular weight of PEGMA–PDMAEA–PCL was calculated to be 18,684 Da.

### 3.2. Hemolysis test

To assess the potential of the nanomaterial *in vivo* application (e.g. intravenous injection), the hemocompatibility was evaluated through hemolysis test (Altmeyer et al., 2016). As shown in Figure 3(D), complete hemolysis of RBCs was observed in water with no intact erythrocytes whereas that treated with PBS had no obvious changes. PEGMA–PDMAEA–PCL micelles exhibited negligible hemolysis capacity to RBCs at various concentrations ranging from 0.05 to 2 mg/mL. The result revealed that the PEGMA–PDMAEA–PCL micelles own appropriate hemocompatibility and may be hopeful as a drug carrier.

### 3.3. Characterization of Que-MMICs

**Figure 4.**
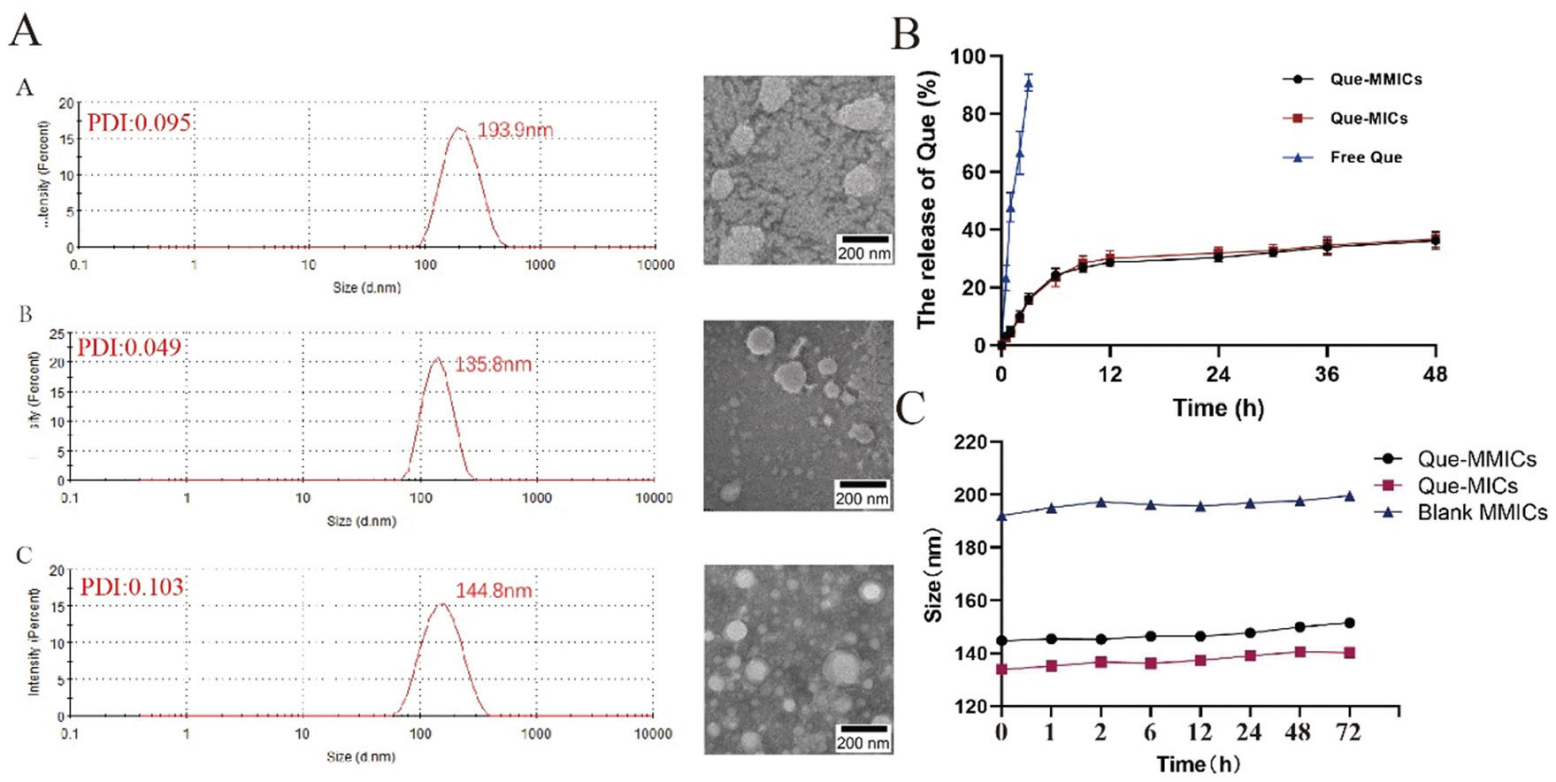
Size and TEM images of (A) blank MICs, Que-MICs, and Que-MMICs. (B) Release profiles of Que formulations at pH 7.4 PBS under 37 °C (*n* = 3). (C) Size change of micelles at room temperature (25 °C) over time (*n* = 3).

The amphiphilic copolymer was comprised of the hydrophilic PEGMA and PDMAEA, and hydrophobic PCL, which could self-assemble into micelles at physiological conditions. Water-insoluble antitumor drug Que was entrapped into the core through hydrophobic interactions between PCL and Que. To further improve targeting ability, biotin was then introduced into micelles. The physicochemical characteristics of nanomedicines have been proven to show significant influence on their biodistribution and therapeutic efficiency *in vivo* (Peng et al., 2017). Hence, diameter, morphology, EE and drug release behavior of Que-MMICs were investigated (Table 1 and Figure 4). Blank micelles showed a size of 193.9 nm (PDI of 0.095) with a narrow size distribution. The entrapment of Que significantly decreased the diameter of Que-MMICs and Que-MICs with PDI of 0.049, which might be attributed to stronger interactions between Que and PCL segments than PCL themselves. Likewise, Que-MMICs demonstrated particle size of 144.8 nm (PDI = 0.103) which was comparable to that of Que-MICs. TEM images revealed the homogeneous size distribution and spherical appearance without no obvious aggregation, which was in accordance with DLS results (Figure 4(A)). A broad consensus has been reached that nanoparticles with diameter ranging from 50 to 200 nm demonstrated a higher accumulation in tumor region after intravenous administration through EPR effect (Kulkarni & Feng, 2013; Li et al., 2017). These suggested that Que-MMICs may effectively accumulated into tumor site after intravenous administration.

**Table 1.** Characterizations of MICs, Que-MICs, and Que-MMICs.

Formulations	Size (nm)	PDI	EE (%)	DL (%)
Blank MICs	193.9	0.095		
Que-MICs	135.8	0.049	68.7	6.43
Que-MMICs	144.8	0.103	85.8	7.90

Low solubility of Que hinders its pharmacological potential and the entrapment into nanoparticles can improve its solubility and stability (Khan et al., 2021). The EE and DL were determined using UV–vis spectrophotometry as previously described (Kumar et al., 2009). As shown in Table 1, DL of Que-MICs and Que-MMICs was 6.43% and 7.90% while EE was 68.7% and 85.8%, respectively, with satisfactory reproducibility (Table 1). The hydrophobic effect is a major driving force behind drug-loading capacity and efficiency especially for hydrophobic drugs (Wang et al., 2007). The hydrophobic interaction between Que and DSPE was greater than that between Que and PCL, which may explain a higher drug loading capacity of Que-MMICs compared to Que-MICs (Mu et al., 2010). *In vitro* drug release behavior of both micelles in PBS was investigated using dialysis method and the result is shown in Figure 4(B). Free Que showed rapid release and almost complete release within 4 h. In contrast, Que release from Que-MICs and Que-MMICs was in a controlled and sustained manner, which was quickly increased in the first few hours and thereafter slowly released. This is due to the hydrophobic core of the block copolymer and the strong interaction between Que and PCL, which can delay the diffusion of the drugs from the core (Yoo & Park, 2001). The results suggested that Que was entrapped well in the hydrophobic core rather than the surface of mixed micelles, which would benefit *in vivo* applications. Moreover, the particle diameter was measured at different intervals. The micelles showed excellent stability without significant change in particle size within 72 h.

### 3.4. *In vitro* cellular uptake evaluation

**Figure 5.**
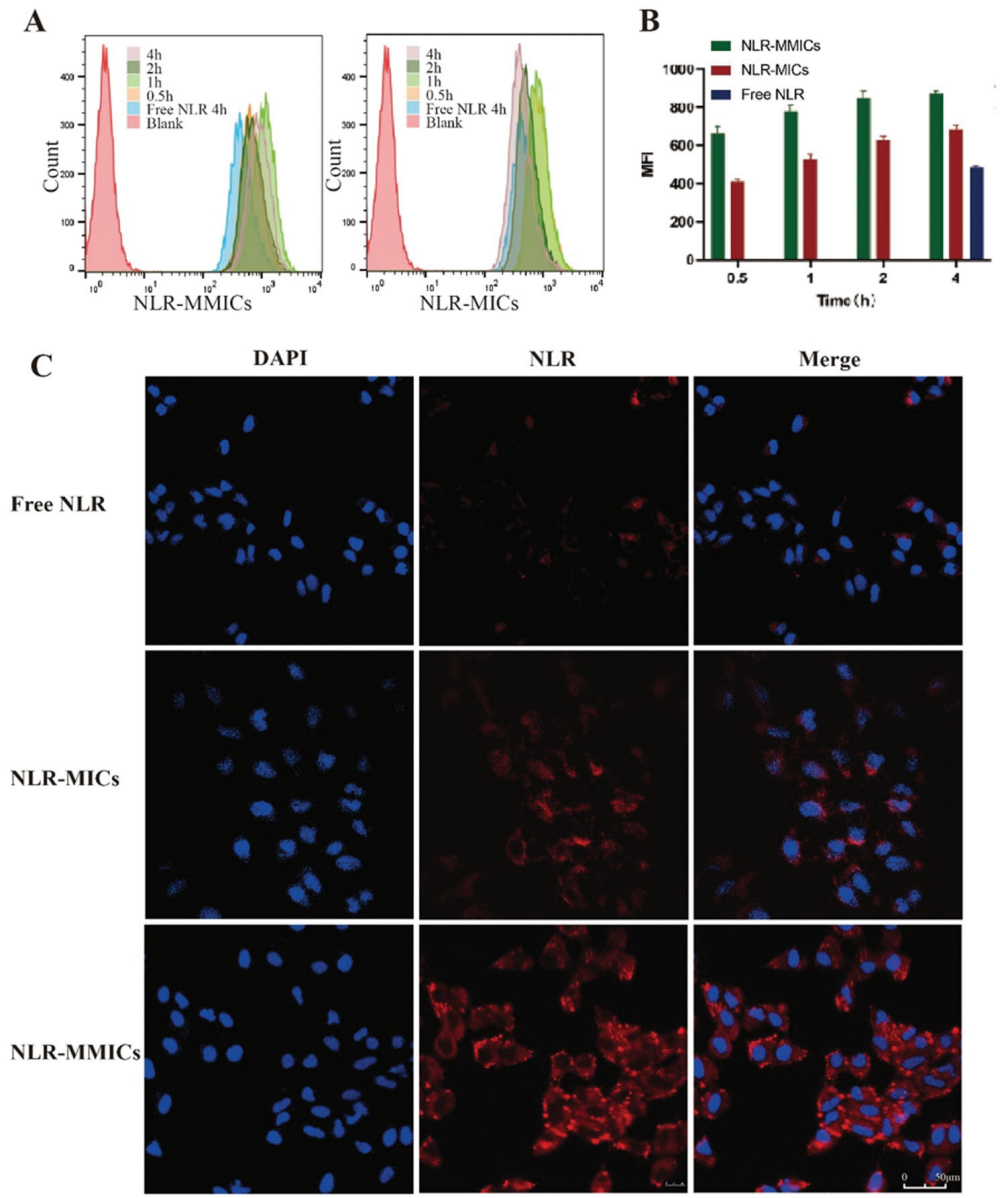
Flow cytometry and CLSM of NLR-MMICs in A549 cells. Flow cytometry (A) and histogram analysis (B) of free NLR, NLR-MICs and NLR-MMICs in A549 cells after incubation for different times. (C) CLSM observation of A549 cells after 4 h incubation with free NLR, NLR-MICs and NLR-MMICs, scale bar = 50 μm.

In general, therapeutic effects of nanomedicine were dependent on their internalization and intracellular release in cancer cells (Behroozi et al., 2018). NLR, as a fluorescence probe, was used to replace Que entrapped in the micelles to investigate their internalization in cancer cells. Cellular uptake in A549 cells was evaluated through flow cytometry and CLSM (Figure 5). NLR-MICs and NLR-MMICs internalization in A549 cells was significantly higher than that of free NLR after 4 h incubation. Interestingly, fluorescence intensity attributed to NLR gradually increased by extending incubation time from 0.5 to 4 h, suggesting that both micelles could be efficiently taken up in a time-dependent manner. Previously, we found that the introduction of ligands could enhance the uptake of particles in corresponding receptors positive cells (Guo et al., 2016). Biotin conjugation significantly improved 1.2-fold cellular uptake compared to NLR-MICs, which was in accordance with previous report (Maiti et al., 2013). To clarify the cellular internalization process and the intracellular trafficking, colocalization experiments were conducted in A549 cells using CLSM. After 4 h incubation, fluorescence intensity of NLR-MICs and NLR-MMICs in A549 cells presented a significant increase compared to free NLR, indicating that the entrapment into micelles overcome cellular barriers and enter intracellular regions. For over-expressed biotin receptors on A549 cell membrane, NLR-MMICs showed an enhanced internalization and intracellular distribution compared to NLR-MICs, which agreed with the results obtained from flow cytometry. Therefore, it was reasonably speculated that Que-loaded nanoparticles could be efficiently internalized into A549 cells via the biotin receptor-mediated endocytosis.

### 3.5. *In vitro* cytotoxicity

**Figure 6.**
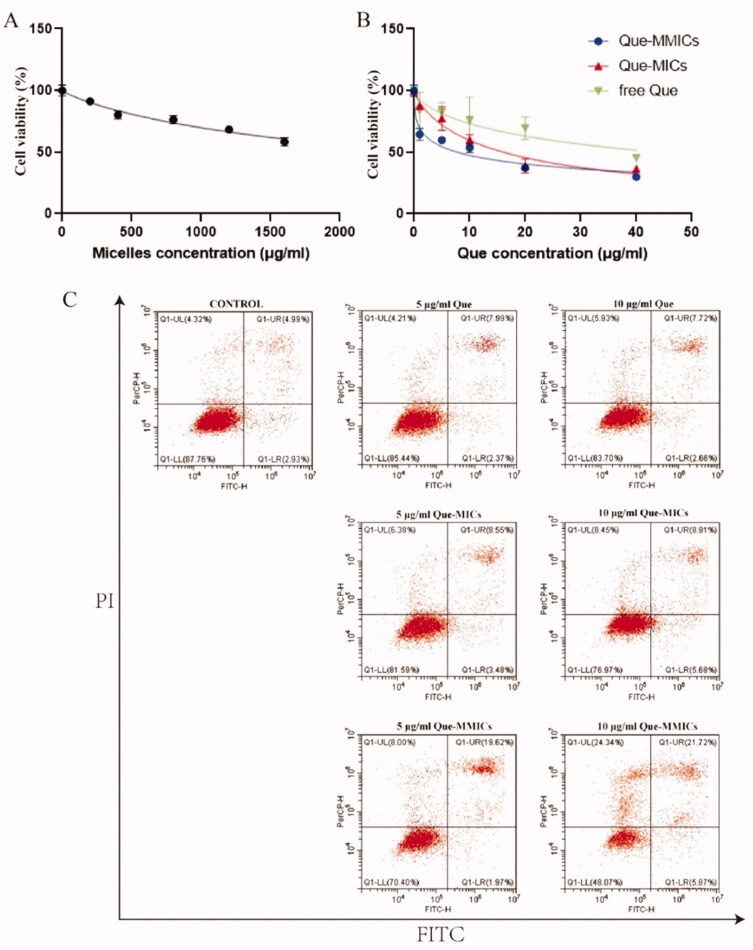
Cell viability of A549 cell after Que formulations treatment. The cytotoxicity of blank micelles (A) and Que formulations (B) for 48 h (*n* = 6). (C) The apoptotic assay was conducted using annexin V/propidium iodide (AV/PI) staining and flow cytometry after A549 cells treated with Que formulations for 24 h.

MTT assay was used to estimate the cytotoxicity of these Que formulations against A549 cells. As shown in Figure 6(B), free Que exhibited weak inhibition effects on the proliferation of A549 cells in a dosed-dependent manner. As a promising adjuvant therapeutic agent, Que has been extensively utilized to inhibit tumor growth through modulating apoptosis, cell cycle, autophagy, and immune response (Guo et al., 2021). Nevertheless, the direct application of Que as a pharmaceutical is obstructed as poor accumulation in tumor and cellular uptake, though it has been in early-stage clinical trials decades ago (Hu et al., 2017). After Que entrapment into both micelles, the viability of A549 cells significantly decreased. Furthermore, biotin modification increased the cytotoxicity of Que as improvements in cellular uptake in A549 cells. The IC_50_ values were also calculated and shown in Table 2. Que-MICs and Que-MMICs exhibited remarkable antiproliferative activity with IC_50_ values 16.15 and 7.83 μg/mL, respectively. Wang et al. designed and synthesized a novel amphiphilic chitosan, self-assembled to load QCT as nanomicelles (Wang et al., 2019). The IC50 was 25 μg/mL and significantly higher than Que-MICs and Que-MMICs. Meanwhile blank micelles exhibited negligible cytotoxicity beyond the concentration studied (Figure 6(A)), indicating the safety of PEGMA–PDMAEA–PCL as a vehicle. These results confirmed that inhibitory effects of Que-MMICs resulted from enhanced internalization of Que, which was in accordance with results obtained from cellular uptake assay.

**Table 2.** IC_50_ values of Que and formulation in A549 cells.

Formulations	IC_50_ (μg/mL)
Free Que	44.22
Que-MICs	16.15
Que-MMICs	7.83

### 3.6. Cell apoptosis assay

It has been reported that Que treatment could induce the apoptosis of tumor cells through activation of the extrinsic death receptor mediated and intrinsic mitochondrial apoptotic pathways (Kedhari Sundaram et al., 2019; Teekaraman et al., 2019). Thus, apoptosis induction of Que and formulations was studied in A549 cells and the results are shown in Figure 6(C). Treatment with free Que achieved 10.36% and 10.38% apoptosis rates at concentration of 5 μg/mL and 10 μg/mL, as compared to control group of 7.96%. Que-MICs induced 12.03% and 14.59% apoptosis of A549 cells at concentration of while 5 μg/mL and 10 μg/mL, respectively. Biotin conjugation further promoted 1.8-fold and 1.9-fold in apoptotic percentage at different Que concentrations. These results were in agreement with cytotoxicity results, which was due to higher cellular uptake of Que-MMICs in A549 cells.

### 3.7. Cell cycle arrest

**Figure 7.**
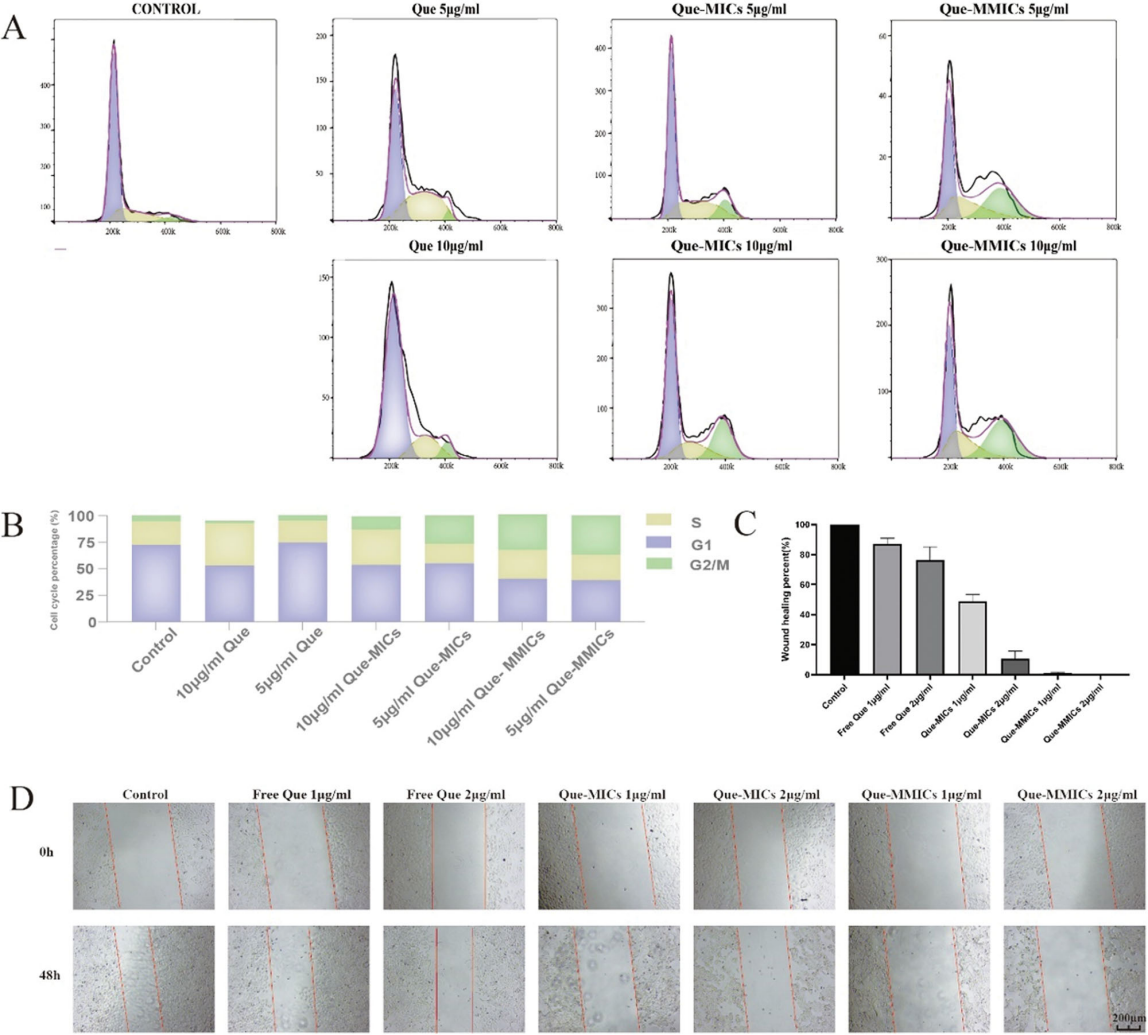
(A) Cell cycle analysis in A549 cells after incubation with different formulations (5 and 10 µg/mL) for 24 h. (B) Histograms display the percentage of cell cycle distribution after treatment with different formulations. (C) The graph shows the quantitative effect. Migration was quantified by measuring the gap closure at the indicated times. Data are represented as mean ± SEM of three independent experiments. (D) A548 cells in a monolayer were wounded, and cells treated with different formulations and at 48 h were photographed. The red lines define the areas lacking cells, scale bar = 200 µm.

Que can induce G2/M cell cycle arrest in numerous cancer cell lines through the regulation of cycle related molecules such as cyclinB1, MiR-34a, and p53 (Lou et al., 2015). In addition, Que displays a high binding affinity to CDK6 and downregulates its expression in A549, arresting cell cycle and reducing cell viability and colony formation (Yousuf et al., 2020). In this study, free Que treatment showed a slight arrest in G2/M, about 2.84% and 5.28% at concentration of 5 and 10 µg/mL, respectively. As shown in Figure 7, A549 cells were evidently arrested in G2/M phase by the treatment of Que-MICs and Que-MMICs in a concentration-dependent manner. For example, it was clearly observed that the percentage of A549 cells in G2/M phase increased from 5.51% to 36.7% after treatment with Que-MMICs at 10 µg/mL, which were obviously more potent than Que-MICs. These data demonstrated that Que-MMICs could effectively inhibit A549 cells through apoptosis induction and cell cycle arrest, higher than that of Que-MICs and free Que, which resulted from efficient encapsulation and high cellular uptake mediated by biotin conjugation.

### 3.8. Migratory capabilities assay

Cell migration is important factor in invasion and metastasis of NSLCs tumor. Recent study showed that Que could inhibit the main cytoskeletal elements, including microfilaments, microtubules and vimentin intermediate filaments, and cytoskeleton-driven processes in A549 cells (Klimaszewska-Wiśniewska et al., 2017). Wound healing assay was then conducted to assess the potential anti-migration activity of different Que formulations. As shown in Figure 7(D), untreated tumor cells demonstrated high mobile capability and almost filled wounded area within 48 h. In contrast, Que-MICs and Que-MMICs significantly inhibited A549 migration in a dose-dependent manner. Figure 7(C) shows that A549 cells receiving 1 μg/mL and 2 μg/mL doses of Que-MMICs exhibited lower wound healing percent (0.99% and 0.35%) than free Que (87.1% and 76.2%) and Que-MICs (48.8% and 10.8%), suggesting the anti-migratory capacity of Que-MMICs to A549 cells.

### 3.9. *In vivo* biodistribution

**Figure 8.**
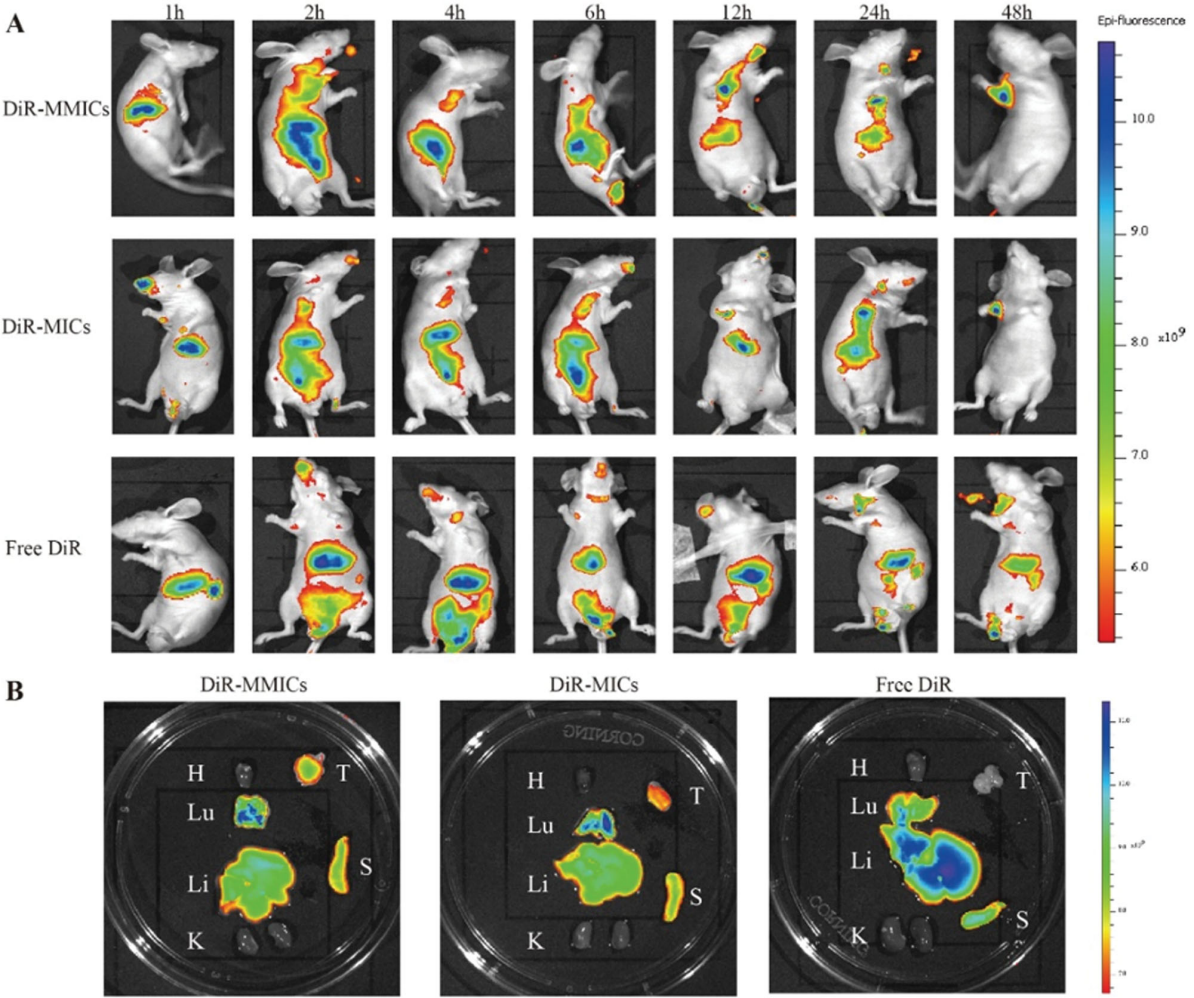
*In vivo* biodistribution of DiR-labeled mixed micelles in A549 inoculation mice after intravenous administration. (A) Fluorescence images of tumor-bearing mice at different times after administration of DiR-MMICs, DiR-MICs, and free DiR. (B) Fluorescence images of excised major organs and tumor tissue after mice sacrifice. T: tumor; H: heart; Lu: lung; Li: liver; S: spleen; K: kidney.

It has been well known that nano-scaled carriers could highly accumulate in solid tumors by the leaky tumor vessels and lack of effective tumor lymphatic drainage termed as EPR effect (Gaucher et al., 2005). Biotinylated nanoparticles are favorably distributed tumor sites as overexpressed biotin receptors on rapidly proliferation malignant cells (Tang et al., 2020). The biodistribution and tumor-targeting efficiency of mixed micelles were evaluated using a noninvasive near-infrared optical imaging technique. As shown in Figure 8, both micelles exhibited a fast and prolonged accumulation in tumor sites. At the first few hours, DiR-MMICs and DiR-MICs were mainly distributed in the liver. Subsequently, fluorescence signals in the tumor site gradually increased over time. After administration for 12 h, DiR-MMICs demonstrated much higher accumulation in tumors over in liver and spleen in comparison DiR-MICs meanwhile no other major organs seemed to be affected. The excised tumors and major organs were observed at 48 h post-injection (Figure 8(B)), and Que-MMICs demonstrated much higher tumor accumulation and lower distribution in liver and spleen than DiR-MICs. These results were consistent with *in vitro* cellular uptake and cytotoxicity assay, indicating that biotin-modified mixed micelles can be used as a highly efficient drug carrier to reach tumor tissue.

### 3.10. *In vivo* antitumor activity

**Figure 9.**
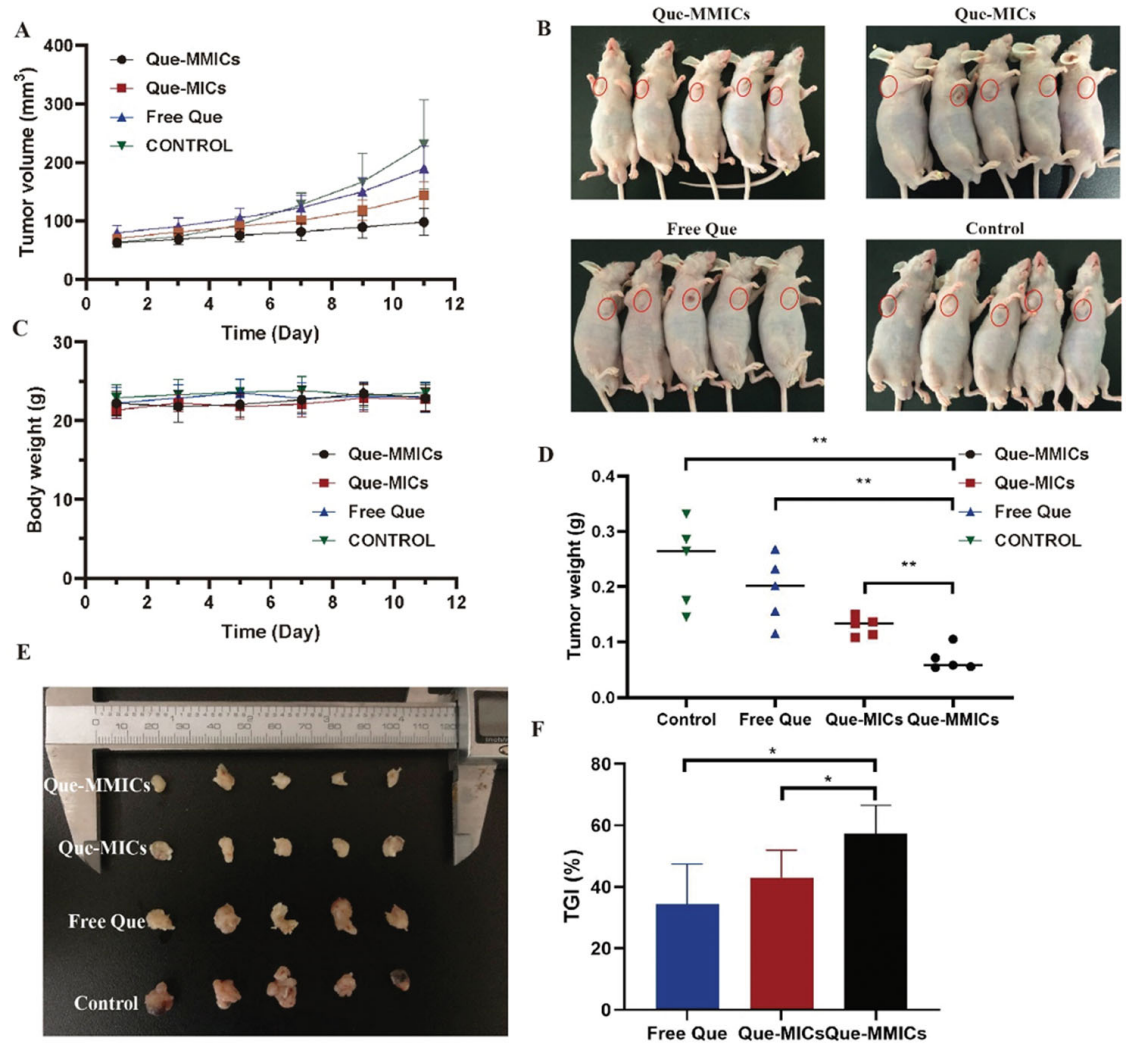
Tumor size (A) and tumor weight (D) of the A549 cells xenograft tumor-bearing nude mice (*n* = 5) after systemic treatment by saline, Que-MICs, Que-MMICs, and free Que (Que = 8 mg/kg). Photograph (B) and body weight (C) of the A549 xenograft tumor-bearing nude mice in each group. Tumors are indicated by ellipses. (E) Images of excised tumors form mice on the 11th day after treatments. (F) Histogram analysis of TGI. *p<.05, **p<.01.

Due to the potent *in vitro* anti-proliferative activity and *in vivo* tumor targeting efficiency of Que-loaded micelles, we have the reason to believe that Que-MMICs can efficiently accumulate into tumors and suppress their growth. Herein, the antitumor activities of these Que formulations were evaluated in A549 tumor xenograft model established in nude BALB/c mice. Figure 9(A) depicts the growth curves of A549 tumors in mice receiving different treatment. It showed that the tumor sizes in control group displayed a rapid and time-dependent increase, which reached 230.7 mm^3^ on 11th days. The administration of free Que resulted in a mean tumor volume of 189.9 mm^3^ and TGI of 34.4%. On the contrary, the mice received Que-MICs (8 mg/kg) show smaller tumor volumes and slower tumor growth rates than free Que group, which achieved a mean tumor volume of only 144.3 mm^3^ and TGI of 43.0% on the 11th day. Encouragingly, biotin modification further reduced tumor volumes compared to non-biotin group, leading to mean tumor volume of 98.2 mm^3^ and TGI of 56.7% (Figure 9(F)). As displayed in Figure 9(B,E), tumors excised from mice receiving various treatments can also illustrate the distinct tumor suppression effects as visual evidences. The exciting *in vivo* antitumor efficacy of Que-MMICs over that of Que-MICs and free Que could be ascribed to their higher tumor targeting efficiency through the combination of EPR effect and active targeting. Moreover, there were no significant differences in body weight of all groups during treatment period, indicating negligible acute toxicities and good safety (Figure 9(C)).

### 3.11. ICH and HE assay

**Figure 10.**
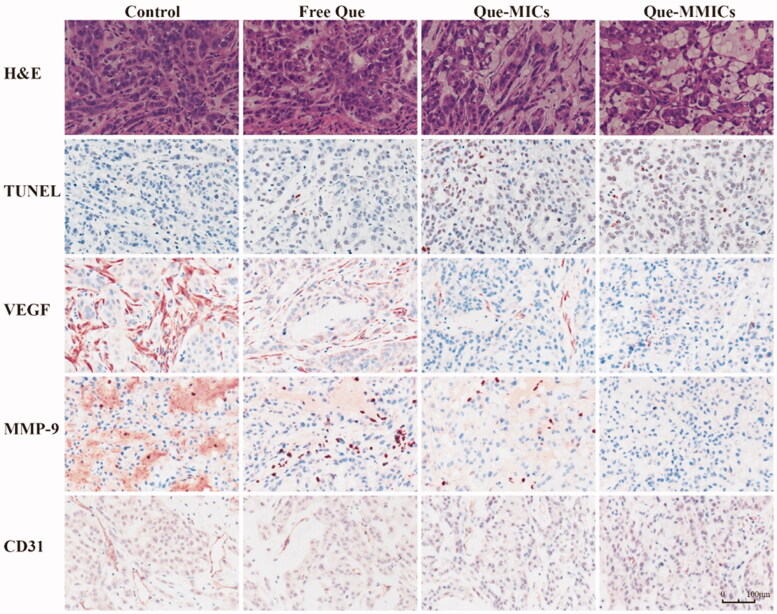
Slices of tumor tissues were generated from each tumor for IHC and H&E analysis. Representative image for H&E, CD31, VEGF, MMP-9 and TUNEL assay are shown. Images were captured with a ×20 objective on a light microscope. The tumors were harvested from the mice 11 days after treatment, scale bar = 100 μm.

To evaluate the histopathological changes in tumors and main organs after different formulations treatment, H&E and ICH analyses were conducted and the results are shown in Figure 10. H&E staining of representative tumor sections in the control group showed aggressive growth with numerous mitotic cells in different stages. Tumors received Que-MMICs treatment showed the lowest proliferation where abundant apoptotic cells showed dense nuclear pyknosis and cytoplasmic karyorrhexis. TUNEL staining was conducted to examine the apoptosis level in the tumors from different groups. The percentage of TUNEL-positive cells is significantly higher in tumors received Que-MMICs treatment compared with that in Que-MICs and free Que groups, which result from more efficient tumor-specific delivery of Que. Hence, H&E and TUNEL staining results further corroborated the superior antitumor efficacy of Que-MMICs than Que-MICs and free Que.

Sustained angiogenesis is one of the central hallmarks of cancer and has been validated as a key target for cancer therapy (Hanahan & Weinberg, 2011). Recent studies have high-lighted that Que could reduce angiogenesis and tumor invasion and metastasis *in vitro* and *in vivo* (Pratheeshkumar et al., 2012). In this process, vascular endothelial growth factor (VEGF) and matrix MMP are key proangiogenic mediators (Quintero-Fabian et al., 2019). VEGF expression in control and free Que groups exhibited well-developed networks of capillaries or small blood vessels around tumors. On the contrast, the Que-MMICs treatment groups displayed severely distorted blood vessels or absence of VEGF positive stains, suggesting potential vascular disrupting properties of Que-MMICs. Consistent with the negative regulation of VEGF gene expression, we also observed a significant downregulation of MMP-9, in response to Que-MMICs treatment. These reductions might be attributed to the suppression of STAT3 activation by Que (Cao et al., 2014). Vessel density was determined by immunolabeling of CD31, which is used primarily to demonstrate the presence of endothelial cells in histological tissue sections. Que-MMICs and Que-MICs apparently reduced the number of vessels compared with free Que or control group. These data demonstrated that Que-MMICs could reduce angiogenesis through VEGF and MMP9 downregulation, which was promising to decrease invasion and metastasis in NSCLC.

### 3.12. Safety evaluation

**Figure 11.**
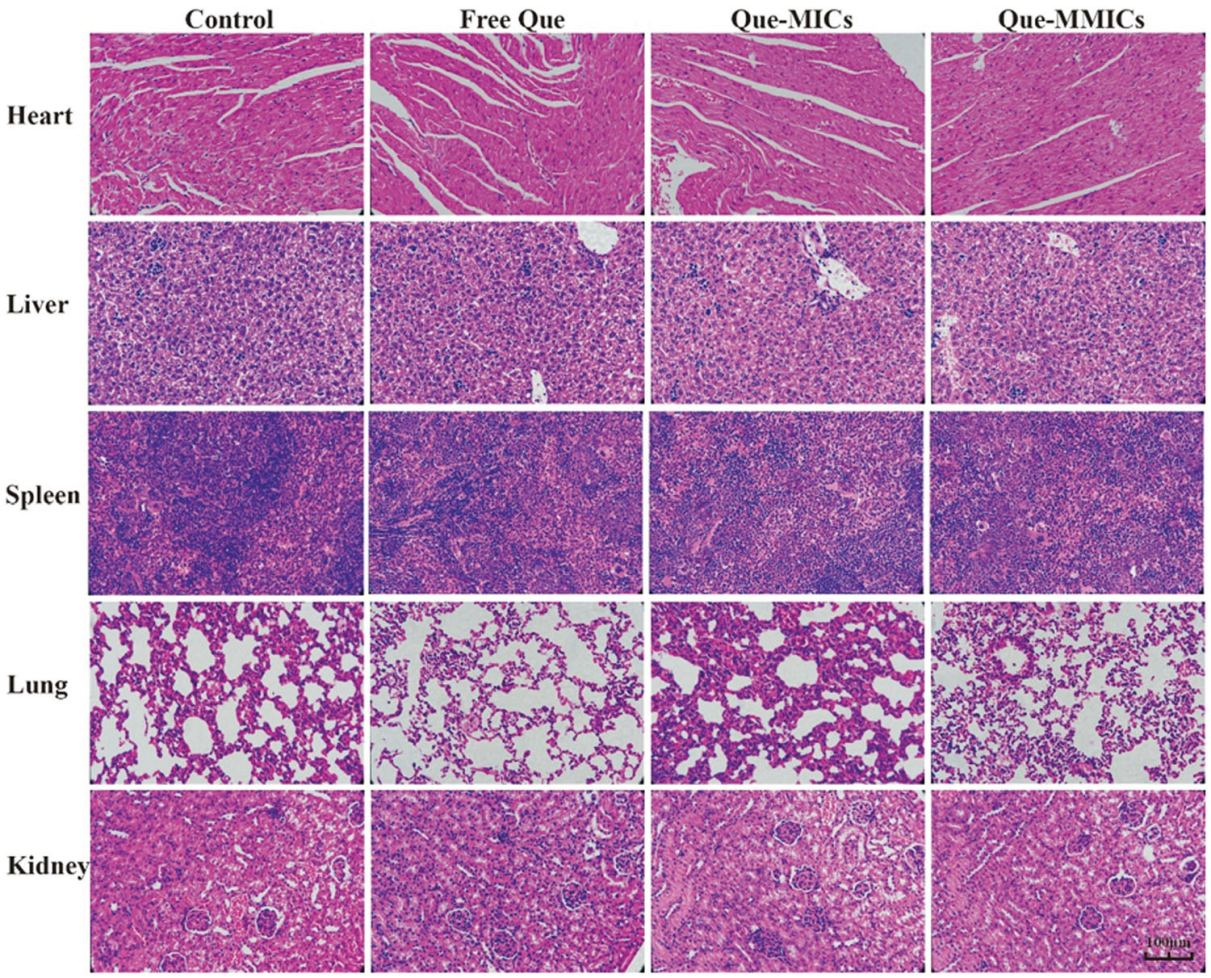
Histological analysis of main organs collected from mice that received different treatments, scale bar = 100 μm.

H&E stains revealed no apparent histological changes in main organs obtained from treatment groups, which were consistent with body weight changes of mice. These confirmed that Que-MMICs could efficiently inhibit the progression of tumor cells and had good safety at dosage up to 8 mg/kg in mice (Figure 11).

## 4. Conclusions

In this paper, we have successfully developed Que-loaded MMICs with biotin conjugation to effectively target biotin-positive A549 cells. Mixed micelles could easily be prepared by co-assembling of the two above-mentioned block copolymers with similar structures; one containing acrylate end groups and the other with biotin end group. The delivery system demonstrated good performance in entrapment of water-insoluble Que and cellular uptake. Moreover, Que-MMICs were investigated for anti-tumor activity *in vitro* and *in vivo*. Que-MMICs displayed the efficient anti-proliferation activity through apoptotic induction and cell cycle arrest *in vitro*. Furthermore, biotin modified micelles demonstrated good tumor-targeting and antitumor effects which were observably more potent than non-targeted micelles and free Que. This was attributed to the introduction of biotin (DSPE–PEG–biotin), facilitating the accumulation of drugs in the tumor via the specific receptor-mediated endocytosis. Collectively, Que-MMICs showed robust *in vitro* and *in vivo* anti-tumor efficacy against A549 cells, paving the way for its further development as a promising drug delivery system.
